# Contextual cues shape facial emotion recognition: a combined behavioral and ERP study

**DOI:** 10.3389/fnins.2025.1710208

**Published:** 2026-01-14

**Authors:** Mónica Toro, Cristian Cortés-Rivera, Francisco Cerić, Juan Carlos Oliveros

**Affiliations:** 1 Laboratorio de Neurociencia Afectiva (LaNA), Instituto de Bienestar Socioemocional (IBEM), Universidad del Desarrollo, Santiago, Chile; 2 Doctorado en Ciencias del Desarrollo y Psicopatología, Universidad del Desarrollo, Santiago, Chile; 3 Department of Psychology, Universidad Católica del Maule, Talca, Chile; 4 The Neuropsychology and Cognitive Neurosciences Research Center (CINPSI Neurocog), Faculty of Health Sciences, Universidad Católica del Maule, Talca, Chile

**Keywords:** affective neuroscience, contextual cues, contextual modulation, emotion recognition, emotional processing, emotional valence, event-related potentials (ERP)

## Abstract

**Introduction:**

Being able to recognize the emotions in others is fundamental to social interaction, yet the precise temporal dynamics by which the brain integrates contextual cues with facial expressions remain unclear. This study used behavioral measures and event-related potentials (ERPs) to investigate how contextual congruency and emotional valence modulate facial emotion recognition in a neurotypical population.

**Methods:**

Participants viewed emotional faces preceded by either congruent or incongruent bimodal cues, combining vocalizations and visual images.

**Results:**

Behaviorally, participants responded faster and made fewer errors during congruent trials than in incongruent trials, indicating that context facilitates emotional processing. At the neural level, incongruent cues elicited a significantly larger P1 component, suggesting that the brain allocates increased early attentional resources to conflicting stimuli. Furthermore, the P3 component was significantly larger for negative stimuli compared to neutral ones, highlighting the role of emotional valence in later stages of cognitive processing.

**Discussion:**

Together, these findings support a multi-stage model of emotional integration, where contextual incongruency impacts processing from early perceptual encoding to later cognitive evaluation. By integrating behavioral and neural evidence, this study clarifies the temporal course of contextual integration in multisensory emotion perception and provides new insights with implications for clinical and applied research.

## 1 Introduction

Recognizing emotions from facial expressions is a cornerstone of human social communication. Facial cues convey critical affective information that enables individuals to interpret others’ internal states, regulate interpersonal interactions, and adapt behavior to rapidly changing contexts (van Kleef and Côté, 2022). Impairments in emotion recognition are strongly associated with social dysfunction, as demonstrated in individuals with autism (Loth et al., 2018), schizophrenia (Gao et al., 2021), traumatic brain injury (Rigon et al., 2018), Parkinson’s disease (Argaud et al., 2018), among other groups, where difficulties in recognizing facial emotions significantly contribute to interpersonal challenges. Beyond clinical populations, accurate and rapid detection of emotional expressions is fundamental for adaptive behavior, guiding decision-making, supporting cooperation, and enabling efficient threat detection. For example, anger can be recognized within a few hundred milliseconds, a speed that provides evolutionary benefits in potentially dangerous situations (Martinez et al., 2016). These processes reflect the capacity of emotions to activate motivational systems that orient physiological and behavioral responses toward adaptive outcomes (Beall and Tracy, 2017).

Although faces are central for affective communication, human emotion perception is an inherently multisensory process influenced by a rich array of contextual cues (de Gelr and Vroomen, 2000; Meyer and Noppeney, 2011). The brain rarely processes an emotional face in isolation. Instead, it integrates visual information with auditory cues (e.g., vocalizations, prosody), body language, and the overall situation to construct a complete understanding of an individual’s emotional state. This contextual integration is crucial, as a single facial expression can be ambiguous or take on different meanings depending on the surrounding information (Barrett and Simmons, 2015). The brain’s ability to efficiently integrate these cues is a key aspect of social cognition. When contextual information is consistent with a facial expression, it facilitates and speeds up emotion recognition. Conversely, when cues are inconsistent, they can lead to an emotional conflict that must be resolved, consuming greater cognitive resources (Puffet and Rigoulot, 2025).

Research over the past decades has demonstrated that facial emotion recognition is not solely determined by facial features themselves, but is systematically shaped by the broader situational context in which emotions are perceived (Aviezer et al., 2017; Stewart et al., 2019; Wieser and Brosch, 2012). Foundational behavioral work showed that facial expressions do not uniquely map onto discrete emotional categories and must instead be interpreted in relation to contextual cues (Carroll and Russell, 1996). Subsequent neurocognitive studies revealed that emotion perception depends on supramodal networks that integrate facial, bodily, and situational information (Peelen et al., 2010), and that higher-level conceptual processes play a central role in constructing both perceived and inferred emotional meaning (Skerry and Saxe, 2014). Recent research continues to highlight the impact of context on emotion interpretation, demonstrating that observers rely heavily on situational information when evaluating facial expressions (Goel et al., 2024). Neuroimaging findings further implicate the medial prefrontal cortex as a key region supporting context–emotion integration (Dirupo et al., 2025). Methodological advances—including Bayesian cue-integration models and the application of large language models—have begun to refine theoretical accounts of context-driven emotion perception (Han et al., 2024). Consistent with this work, a recent meta-analysis reported robust influences of both facial cues and contextual information on emotion labeling, with effects modulated by cue congruency and stimulus clarity (Steward et al., 2025).

Electrophysiological studies, particularly those using event-related potentials (ERPs), have provided invaluable insight into the temporal dynamics of this integration process. Early components, such as the P1, a positive wave peaking around 100 ms, are thought to reflect initial sensory and attentional processing (Cortés-Rivera and Cerić, 2020). Research has shown that the P1 amplitude is modulated by attention and salience, with larger responses to stimuli that are particularly relevant or unexpected (Denefrio et al., 2017). In the context of emotion, an increased P1 to incongruent contextual cues suggests that the brain allocates more early-stage attention to resolve conflicting information, highlighting the rapid, automatic nature of this initial conflict detection (Watson et al., 2013).

In addition to these early markers, the N2 component, a negative deflection that peaks approximately 200–350 ms after stimulus onset, is widely recognized as a neurophysiological index of cognitive control and conflict monitoring (Nieuwenhuis et al., 2003). In tasks involving conflicting or ambiguous stimuli, a more negative (larger) N2 amplitude is observed, reflecting the increased neural resources recruited by the brain’s conflict-monitoring system—most notably in the anterior cingulate cortex (ACC). Therefore, in the context of emotional perception, the N2 serves as a marker of the detection of a mismatch between contextual cues and facial expressions, which triggers a greater need for cognitive control to resolve the conflict and ensure an accurate interpretation (Folstein and Van Petten, 2007; Yuan et al., 2007).

Furthermore, later ERP components such as the P3 (a late positive component peaking around 300–600 ms) are associated with higher-order cognitive functions, including stimulus evaluation, memory updating, and attentional allocation to motivationally significant stimuli. The P3 amplitude is highly sensitive to both the arousal and valence of emotional stimuli, typically showing larger amplitudes for more arousing or negative content (Conroy and Polich, 2007; Olofsson et al., 2008). This modulation of P3 amplitude suggests that emotional valence plays a critical role in later, more elaborative stages of cognitive processing. Together, these distinct ERP components provide a neurophysiological window into a multi-stage model of emotional processing, where contextual cues are integrated from early sensory stages to later cognitive evaluative stages.

Despite a growing body of work, the precise temporal course by which the human brain integrates contextual and facial emotional information is still a matter of debate. This study aims to bridge this gap by examining the behavioral and electrophysiological correlates of facial emotion recognition when contextual cues are either congruent or incongruent. Based on the existing literature, we hypothesize that participants will exhibit faster reaction times and higher accuracy in recognizing facial expressions during congruent trials compared to incongruent trials. Furthermore, we predict that this contextual modulation will be reflected in the event-related potentials (ERPs). Specifically, we hypothesize that auditory–visual congruency will modulate the amplitude of the N2 component, with incongruent trials eliciting a larger N2 amplitude compared to congruent trials, reflecting an early stage of conflict detection. We also hypothesize that auditory–visual congruency will modulate the amplitude of the P3 component, with congruent trials showing a larger P3 amplitude, indicating a later stage of cognitive evaluation and context updating. We also hypothesize that the P1 component, an early marker of sensory processing, will be modulated by the emotional valence of the facial stimulus itself, with positive and negative expressions eliciting a larger P1 amplitude compared to neutral expressions. We do not anticipate a significant effect of congruency on the P1 component. Our findings will provide a more detailed understanding of the dynamic interplay between context and emotional perception, with potential implications for clinical research and the development of new interventions for social communication disorders.

## 2 Materials and methods

### 2.1 Participants

A total of 45 individuals were recruited to participate in the experiments conducted at the Affective Neuroscience Laboratory of the Universidad del Desarrollo in Santiago, Chile. After the application of the exclusion criteria, the sample consisted of 39 right-handed healthy adults (19 women, aged 18–40, with a mean age of 27.1; see Table 1). From these participants, six were excluded for the ERP analysis because the collected EEG data did not meet the pre-processing criteria (see the “EEG recordings and processing” section). The final ERP sample consisted of 33 participants (17 women, aged 19–37, with a mean age of 25.1).

**Table 1:** Descriptive statistics for the age of the participants.

Sex	*N*	Mean	Median	*SD*	Minimum	Maximum
Men	20	27.8	27.5	7.01	19	40
Women	19	26.4	24.0	7.21	18	38

Each participant reported having normal hearing and normal or corrected-to-normal vision, and none reported any neurological or psychiatric condition. The criteria for exclusion from each experiment included having a first language other than Spanish, any comorbid conditions such as epilepsy or ADHD, as well as high scores in self-reported measures of mental health. Depression symptoms were evaluated using the BDI-II (Beck et al., 1961; validated in Chile by Valdés et al., 2017) and the PHQ-9 (Kroenke et al., 2001; validated in Chile by Baader et al., 2012). Anxiety symptoms were evaluated using the STAI (Spielberger et al., 1983; validated in Chile by Vera-Villarroel et al., 2007). Finally, the subjects’ current emotional experience was assessed using Positive and Negative Affect Schedule (PANAS; Watson et al., 1988; validated in Chile by Vera-Villarroel et al., 2019).

All participants provided informed written consent after obtaining both a verbal and written explanation of the study. This study was approved by the Ethics Committee at the Universidad del Desarrollo (No. 06112023-MTV) and followed the principles of the Declaration of Helsinki.

### 2.2 Material

Contextual visual stimuli were selected from the International Affective Picture System (IAPS), which has been adapted and validated in Chile (Silva, 2011). This set comprised a total of 119 images. Selection was based on valence ratings, where a score of 8 represents high pleasure (i.e., positive valence) and a score of 0 represents low pleasure (i.e., negative valence). The auditory stimuli consisted of a set of sound stimuli designed to include pseudo-utterances (i.e., nonverbal vocalizations with emotional prosody) produced by two men and two women (amateur actors), based on a previous study (Rigoulot and Pell, 2014). These vocalizations were digitally recorded and subsequently validated by three judges per stimulus with respect to their emotional content. Inter-rater reliability was assessed using intraclass correlation coefficient (ICC) estimates based on a two-way mixed-effects model. ICC values ranged from 0.58 for male sound stimuli (95% CI [0.436, 0.710]) to 0.71 for female sound stimuli (95% CI [0.557, 0.814]). Inter-rater agreement was further examined using Cohen’s kappa, which indicated substantial agreement across judges, ranging from 0.58 for male sound stimuli to 0.73 for female sound stimuli. The final auditory set included 48 pseudo-utterances: 16 positives (*M* = 6.43, *SD* = 1.50), 16 neutrals (*M* = 5.11, *SD* = 1.53), and 16 negatives (*M* = 0.70, *SD* = 0.92), balanced by speaker gender. Regarding facial expressions, a set of previously standardized facial stimuli was used, including expressions of happiness/joy (positive valence), anger/sadness (negative valence), and neutrality (Ceric, 2008). The emotional face stimuli featured Chilean individuals aged between 18 and 40 years. This set included 32 photographs of positive-valence faces (15 women), 35 neutral-valence faces (17 women), and 35 negative-valence faces (17 women). Following the recommendations of Fujimura and Umemura (2018), all facial stimuli exceeded chance levels for emotional categorization (>49% of ratings) and confidence scores (>65% of ratings). Gender balance was maintained during stimulus presentation, with 40 randomly selected stimuli per emotional valence condition. All visual stimuli were presented in black and white, with brightness and saturation thresholds adjusted to balance black-and-white contrasts. They were also adjusted to a uniform size and proportion, in accordance with the protocol established by Ceric (2012).

### 2.3 Procedures

**Figure 1:**
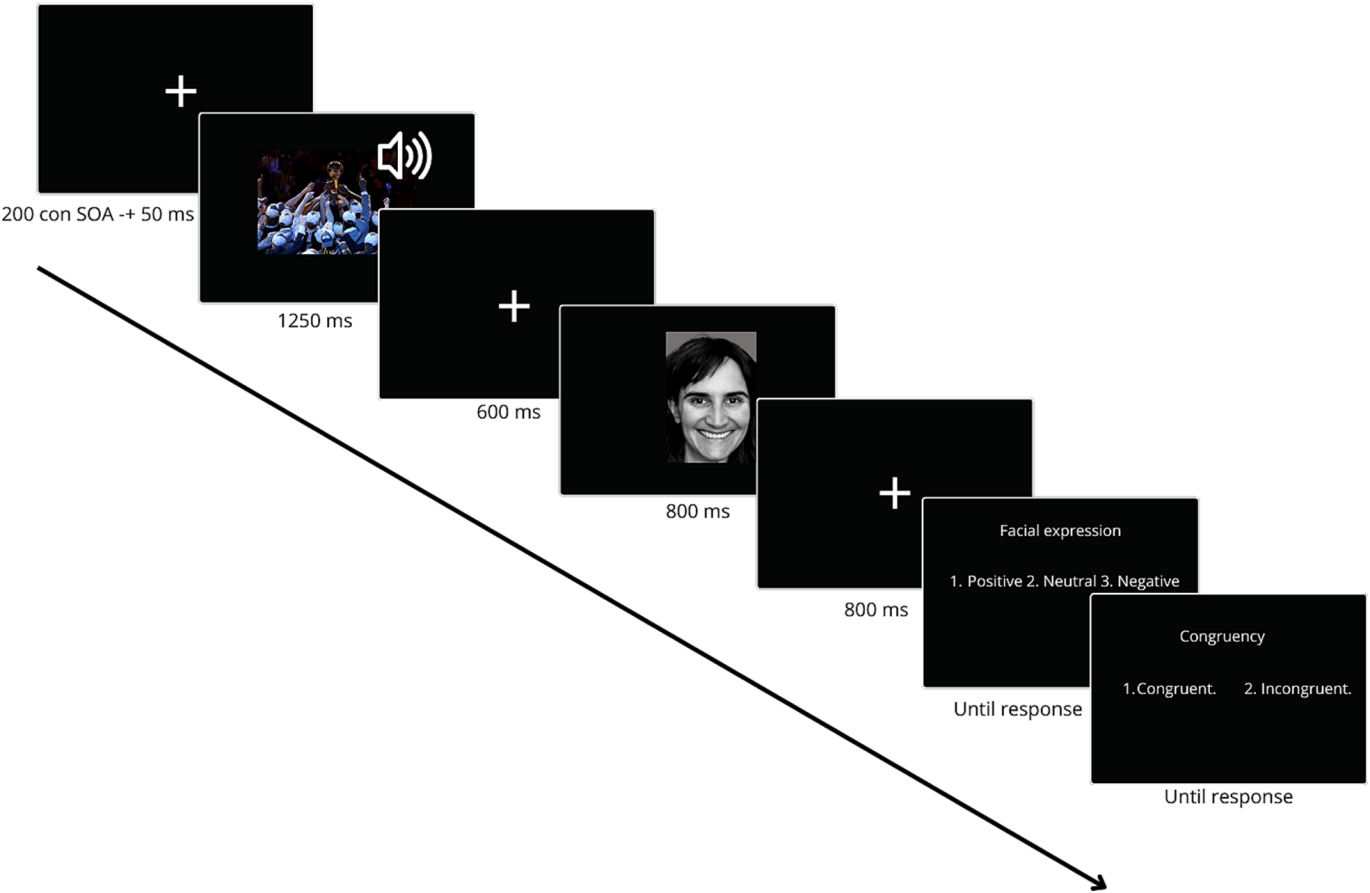
Timeline of a trial from the bimodal emotional congruence attribution task. Each trial began with a congruent bimodal stimulus (contextual image and pseudo-utterance) presented for 1,250 ms, followed by a fixation cross for 500 ms. An emotional facial expression was then displayed for 800 ms. After another 500 ms fixation, participants answered two questions: One to evaluate the emotional valence of the facial expression (positive, neutral, or negative) and another to assess the congruency between the bimodal cue and the facial expression (congruent or incongruent). A total of 120 trials were conducted in a pseudo-random order.

The procedure was carried out in an environment with regulated temperature and lighting. The experiments were displayed on a 22-inch computer screen. E-Prime 2.0 software (Psychology Software Tools, Pittsburgh, PA, USA) was used to design and present the experimental task. The study examined bimodal emotional congruence attribution, as illustrated in Figure 1.

Before the formal experiment began, participants underwent a practice phase, during which prepared explanations were given to ensure they understood the task. This phase included five trials with random stimuli, identical to those used in the experimental task. Feedback was provided after each trial. During this period, participants could practice the task, review the instructions, and ensure they understood the questions.

Before starting the experimental task, a resting state sequence was performed. This sequence consisted of a fixation cross for 5 min, in which each participant had to remain calm while looking at the fixation cross. After that, they were instructed to remain calm with their eyes closed for another 5 min. Once this was completed, the experiment began.

The experimental sequence consisted of 120 trials in which photographs of faces displaying emotional expressions were presented. These photographs were distributed equally across three valence conditions: positive, neutral, and negative. Each facial expression was preceded by a congruent bimodal stimulus consisting of a contextual image and a pseudo-utterance vocalization (i.e., nonverbal vocalizations with emotional prosody), which were presented simultaneously. Each trial was constructed by simultaneously superimposing a visual stimulus and an auditory stimulus to create this bimodal presentation, that were emotionally congruent but originated from independent sources. When an image originally contained faces, these were pixelated to ensure that participants viewed only the contextual scene. This prevented facial information from influencing their interpretation of the cues.

Each trial contained a stimulus interval ranging from 2,000 to 3,000 milliseconds. At the start of the experiment, a white fixation cross was displayed for 200 ms. The process for each trial is as follows: First, the bimodal stimuli appeared for 1,250 ms. Then, the fixation point “+” appeared for 500 ms, followed by the emotional facial expression for 800 ms. Next, the fixation point “+” appeared again for 500 ms. Finally, two questions were presented. The first one evaluated facial expresión as (1) positive, (2) neutral or (3) negative. And the second question evaluated congruency between the bimodal que and the facial expression as (1) congruent and (2) incongruent.

The emotional stimuli were presented in a pseudo-random order, and participants had no time limit to answer the final questions. At the end of each experiment, a stop signal reaction time (SSRT) was presented, which stopped the recording and ended the experiment. At the end of the experiment, all participants received a healthy snack as compensation after the experiment was finished and a debriefing of the study.

### 2.4 EEG recordings and processing

Electroencephalography (EEG) data was recorded using a 64-channel EEG system (Electrical Geodesics, Inc.). Electrodes were placed according to the 10–20 system and linked to a Net Amps 300 amplifier. The ground electrode was placed in the midline parietal area, and the reference electrode was placed at the vertex. Throughout the session, impedances were kept below 40 kΩ.

EEG data were processed offline using MATLAB software, version 2022b (The MathWorks, Inc., 2022). The process of obtaining the event-related potentials (ERPs) was carried out using the EEGLAB toolbox (version 2022.1; Delorme and Makeig, 2004) and the ERPLAB toolbox (version 12.01; Lopez-Calderon and Luck, 2014). The data were downsampled to 500 Hz and subjected to a 0.1–30 Hz bandpass filter. During an initial examination of the data, segments with voltage readings of ≤0.5 μV or ≥1,500 μV that lasted 200 ms or more were flagged as contaminated by artifacts and removed from further analysis. Participants were excluded from subsequent analyses if the number of bad segments exceeded 30% of the total (Bocincova and Johnson, 2019). The data were divided into 1,000-ms epochs (−200 ms to 800 ms relative to stimulus onset).

The segments were analyzed using Independent Component Analysis (ICA). Following the recommendations of Chaumon et al. (2015), components were evaluated using a combination of topographical maps, time-course characteristics, and power-spectrum profiles to ensure accurate classification and to avoid excessive or unjustified component removal. Components clearly attributable to eye blinks, saccades, or muscle activity were manually identified and removed using semi-automatic ICA inverse process without reverting them to the electrode space. A subsequent review of the data was conducted, and segments with activity levels of ≤0.5 μV or ≥200 μV lasting ≥200 ms were removed. After re-referencing to the common average reference, the data were merged across sessions and reorganized based on emotional valence conditions. The baseline was normalized to the 200 ms preceding stimulus onset. Segments containing solely accurate responses were averaged for each participant in order to perform the ERPs analysis.

The ERPs were time-locked to the presentation of the pictures. The visual P1, N2, and P3 components were identified by visually inspecting both the grand average and the individual averages. Based on previous studies of the multisensory integration of emotions and average ERP waveforms (Chen et al., 2016; Gu et al., 2013; Portnova et al., 2021), we selected parietal and occipital electrodes to analyze the P1 component (100–150 ms), N2 component (160–250 ms), and P3 component (270–340 ms). We obtained the ERP amplitude and latency for each participant by segmenting, superimposing, and averaging the EEG data based on event markers.

### 2.5 Statistical analysis

An *a priori* power analysis was conducted using GPower 3.1 (Faul et al., 2007) to determine the required sample size. The analysis was based on a repeated-measures ANOVA with within-subject factors, an assumed small-to-moderate effect size (*d* = 0.30, corresponding to *f* ≈ 0.15), an alpha level of 0.05, and a desired statistical power of 0.80. The results indicated that a minimum of 30 participants would be required to detect the hypothesized effect. To account for potential data loss due to artifacts or participant exclusion, we aimed to recruit approximately 45 participants.

Descriptive statistics were examined to understand the distribution of sociodemographic data. Parametric tests were used if the data conformed to a normal distribution; otherwise, nonparametric tests were used. For the behavioral analysis, we removed extremely fast and slow reaction times (RTs). Specifically, we excluded RTs shorter than 100 ms, longer than 2,500 ms, and erroneous trials. RTs more than three standard deviations above the participant’s mean were also eliminated. The retained RTs were subjected to a logarithmic transformation prior to statistical analysis to reduce the influence of skewness and approximate a normal distribution. Accuracy (ACC) was calculated as the proportion of correct responses to the total number of trials. For the ERP analysis, a repeated-measures ANOVA was performed with RT, ACC, and ERP mean amplitudes as the factors. Greenhouse–Geisser correction was applied when the sphericity assumption was violated. Significant main effects were followed up with *post hoc* pairwise comparisons adjusted for multiple comparisons using Bonferroni correction. Descriptive statistics, correlations and (Bayes) Factorial ANOVA were analyzed using JASP (version 0.95.1; JASP Team, 2024). Raincloud plots were performed using R (version 4.5.0; R Core Team, 2021).

## 3 Results

### 3.1 Correlations between sociodemographic variables and self-reported measures

Descriptive statistics and Spearman’s rank correlation coefficients for the sociodemographic and self-reported measures are presented in Table 2. The sample showed a mean PHQ-9 score of 4.46 (*SD* = 3.76), a mean PANAS score of 16.97 (*SD* = 11.88), a mean STAI-S score of 27.49 (*SD* = 7.71), and a mean BDI-II score of 6.72 (*SD* = 5.37). The average age was 27.10 years (*SD* = 7.11), with an equal distribution of males and females (*M* = 0.49, *SD* = 0.50). No significant differences were observed in the evaluated variables according to the sex of the individuals (all *p*s > 0.30; see Supplementary material).

**Table 2:** Descriptive statistics and correlations between sociodemographic and self-reported measures.

Variable	*M*	SD	Range	1	2	3	4	5
1. PHQ-9	4.46	3.76	0–13	—	—	—	—	—
2. PANAS	16.97	11.88	−17–38	−0.688***	—	—	—	—
3. STAI-S	27.49	7.71	16–60	0.388*	−0.442**	—	—	—
4. BDI-II	6.72	5.37	0–19	0.627***	−0.622***	0.439**	—	—
5. Age	27.10	7.11	18–40	−0.243	0.263	−0.145	0.051	—
6. Sex	0.49	0.50	0–1	0.071	−0.128	0.155	0.046	−0.119

Correlation coefficients were computed with Spearman’s rank correlation coefficient. M, mean; SD, standard deviation; *p < 0.05; **p < 0.01; ***p < 0.001.

Although our sample size is small and correlations are less stable in small samples (Schönbrodt and Perugini, 2013), reporting these correlations may help motivate future research. Correlation analysis revealed a significant negative association between PHQ-9 and PANAS scores, *r*ₛ = −0.688, *p* < 0.001. PHQ-9 was also positively correlated with both STAI-S, *r*ₛ = 0.388, *p* < 0.05, and BDI-II, *r*ₛ = 0.627, *p* < 0.001. Additionally, PANAS was negatively correlated with STAI-S, *r*ₛ = −0.442, *p* < 0.01, and BDI-II, *r*ₛ = −0.622, *p* < 0.001. A significant positive correlation was observed between STAI-S and BDI-II, *r*ₛ = 0.439, *p* < 0.01. No significant correlations were found between the sociodemographic variables (age and sex) and any of the self-reported measures (*p* > 0.05). These results indicate that, within this sample, the self-reported measures of depression, anxiety, and affect are significantly correlated.

### 3.2 Behavioral results

#### 3.2.1 Reaction times (RTs) data

**Figure 2:**
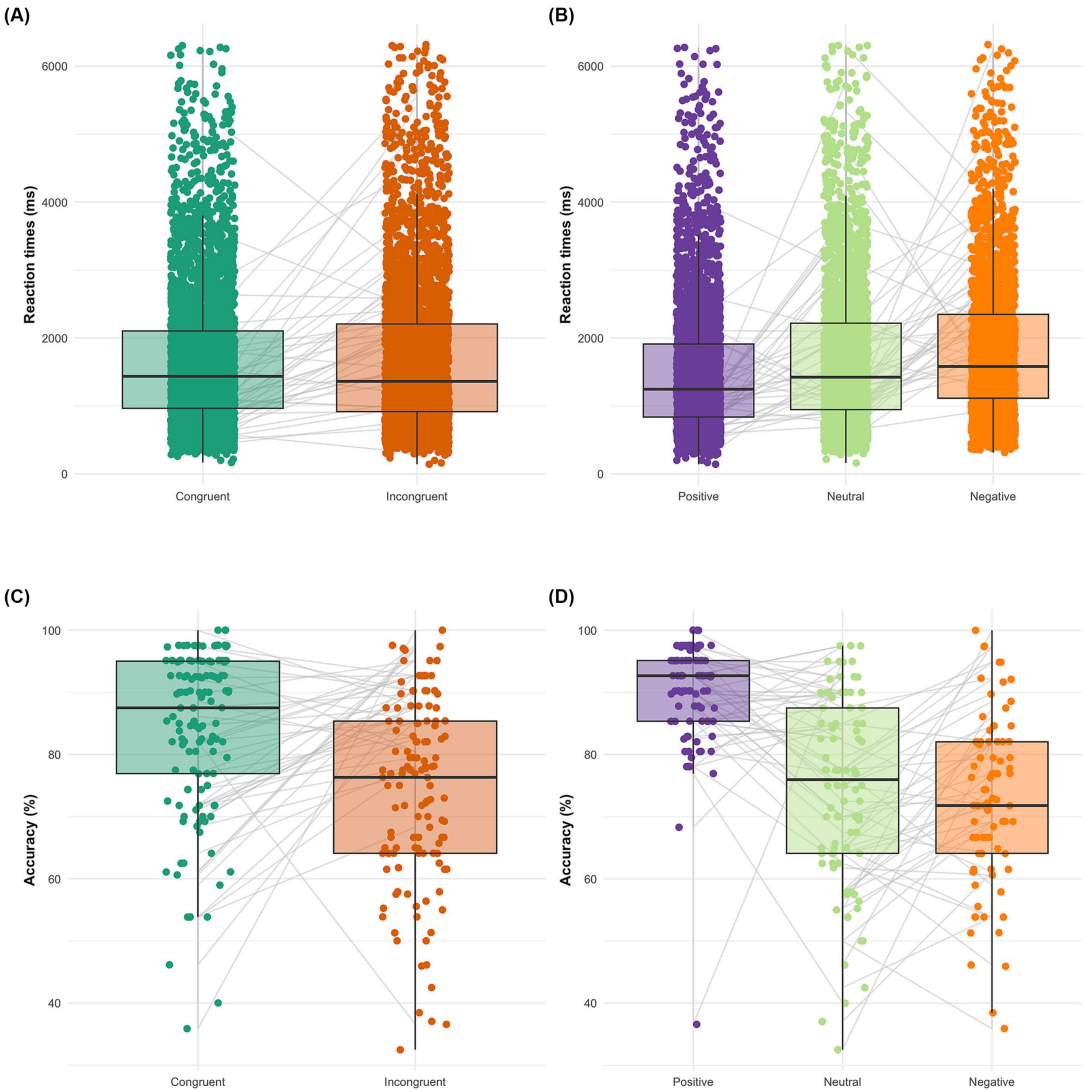
Behavioral performance on the bimodal emotional congruence attribution task. Box plots showing individual reaction times (RTs) for each congruence condition **(A)** and valence condition **(B)**. Box plots showing individual accuracy scores for each congruence condition **(C)** and valence condition **(D)**. The data points for each participant are connected by a line, illustrating the within-subject design.

We conducted a 3 × 2 ANOVA with Emotional Valence (positive, neutral, negative) and Congruency (congruent vs. incongruent) as within-subjects factors. This analysis revealed a significant main effect of Emotional Valence, *F*(2, 4,259) = 98.80, *p* < 0.001, *ηp*^2^ = 0.044. The main effect of Congruency was also significant, *F*(1, 4,259) = 28.66, *p* < 0.001, *ηp*^2^ = 0.007. Importantly, we also found a significant interaction between Emotional Valence and Congruency, *F*(2, 4,259) = 10.84, *p* < 0.001, *ηp*^2^ = 0.005. *Post hoc* comparisons with Bonferroni adjustments were performed to examine this interaction (see Table 3). These analyses revealed that for Positive valence, the congruent condition resulted in faster response (*M* = 7.07, SE = 0.022) than the incongruent condition (*M* = 7.25, SE = 0.018), *t*(4259) = − 5.90, *p* < 0.001, Cohen’s *d* = −0.30, 95% CI [−0.402, −0.201]. Similarly, for Neutral valence, the congruent condition resulted in faster response (*M* = 7.10, SE = 0.029) than the incongruent condition (*M* = 7.24, SE = 0.020), *t*(4259) = −4.37, *p* < 0.001, Cohen’s *d* = −0.25, 95% CI [−0.359, −0.136]. However, for Negative valence that pattern of results was not observed, *t*(4259) = 0.67, *p* = 1.000, Cohen’s *d* = 0.039, 95% CI [−0.0737, 0.151]. Overall, these results indicated that congruency modulated reaction times specifically in trials with positive and neutral valence, but not for negative trials (Figure 2).

**Table 3:** *Post-hoc* comparisons for reaction time.

Conditions	Comparisons	*MD*	*SEM*	*t*	Cohen’s *D*	*p*_Bonf_
Negative congruent	Neutral congruent	0.346	0.036	9.676	0.613	<0.001
	Positive congruent	0.374	0.033	11.305	0.663	<0.001
	Negative incongruent	0.022	0.032	0.673	0.039	1.000
	Neutral incongruent	0.206	0.030	6.798	0.365	<0.001
	Positive incongruent	0.204	0.030	6.760	0.361	<0.001
Neutral congruent	Positive congruent	0.028	0.035	0.813	0.050	1.000
	Negative incongruent	−0.324	0.034	−9.556	−0.574	<0.001
	Neutral incongruent	−0.140	0.032	−4.365	−0.248	<0.001
	Positive incongruent	−0.142	0.032	−4.461	−0.252	<0.001
Positive congruent	Negative incongruent	−0.352	0.031	−11.326	−0.624	<0.001
	Neutral incongruent	−0.168	0.029	−5.786	−0.298	<0.001
	Positive incongruent	−0.170	0.029	−5.903	−0.302	<0.001
Negative incongruent	Neutral incongruent	0.184	0.028	6.551	0.327	<0.001
	Positive incongruent	0.182	0.028	6.512	0.323	<0.001
Neutral incongruent	Positive incongruent	−0.002	0.026	−0.091	−0.004	1.000

Bonferroni corrections were applied for the pairwise comparisons.

#### 3.2.2 Accuracy (ACC) data

We conducted a 3 × 2 ANOVA with Emotional Valence (positive, neutral, negative) and Congruency (congruent vs. incongruent) as within-subjects factors. This analysis revealed a significant main effect of Emotional Valence, *F*(2, 4,259) = 93.83, *p* < 0.001, *ηp*^2^ = 0.042. The main effect of Congruency was also significant, *F*(1, 4,259) = 29.73, *p* < 0.001, *ηp*^2^ = 0.007. Importantly, we also found a significant interaction between Emotional Valence and Congruency, *F*(2, 4,259) = 15.34, *p* < 0.001, *ηp*^2^ = 0.007. Post hoc comparisons with Bonferroni adjustments were performed to examine this interaction (see Table 4). These analyses revealed that for Positive valence, the congruent condition resulted in a more accurate response (*M* = 0.94, SE = 0.010) than the incongruent condition (*M* = 0.82, SE = 0.012), *t*(4259) = 5.69, *p* < 0.001, Cohen’s *d* = 0.29, 95% CI [0.190, 0.391]. Similarly, for Negative valence, the congruent condition resulted in a more accurate response (*M* = 0.82, SE = 0.017) than the incongruent condition (*M* = 0.69, SE = 0.018), *t*(4259) = 5.40, *p* < 0.001, Cohen’s *d* = 0.31, 95% CI [0.197, 0.422]. However, for Neutral valence that pattern of results was not observed, *t*(4259) = −1.40, *p* = 1.000, Cohen’s *d* = −0.080, 95% CI [−0.191, 0.032]. Overall, these results indicated that congruency modulated accuracy specifically in trials with positive and negative valence, but not for neutral trials (Figure 2).

**Table 4:** *Post-hoc* comparisons for accuracy.

Conditions	Comparisons	*MD*	*SEM*	*t*	Cohen’s *D*	*p*_Bonf_
Negative congruent	Neutral congruent	0.168	0.026	6.440	0.408	<0.001
	Positive congruent	−0.121	0.024	−5.018	−0.294	<0.001
	Negative incongruent	0.127	0.024	5.402	0.309	<0.001
	Neutral incongruent	0.135	0.022	6.115	0.328	<0.001
	Positive incongruent	−0.001	0.022	−0.066	−0.004	1.000
Neutral congruent	Positive congruent	−0.289	0.025	−11.429	−0.702	<0.001
	Negative Incongruent	−0.041	0.025	−1.642	−0.099	1.000
	Neutral incongruent	−0.033	0.023	−1.400	−0.079	1.000
	Positive incongruent	−0.169	0.023	−7.287	−0.411	<0.001
Positive congruent	Negative incongruent	0.249	0.023	10.947	0.603	<0.001
	Neutral incongruent	0.256	0.021	12.103	0.623	<0.001
	Positive incongruent	0.120	0.021	5.685	0.291	<0.001
Negative incongruent	Neutral incongruent	0.008	0.021	0.386	0.019	1.000
	Positive incongruent	−0.129	0.020	−6.315	−0.313	<0.001
Neutral incongruent	Positive incongruent	−0.137	0.019	−7.313	−0.332	<0.001

Bonferroni corrections were applied for the pairwise comparisons.

### 3.3 ERP results

#### 3.3.1 P1 component

**Figure 3:**
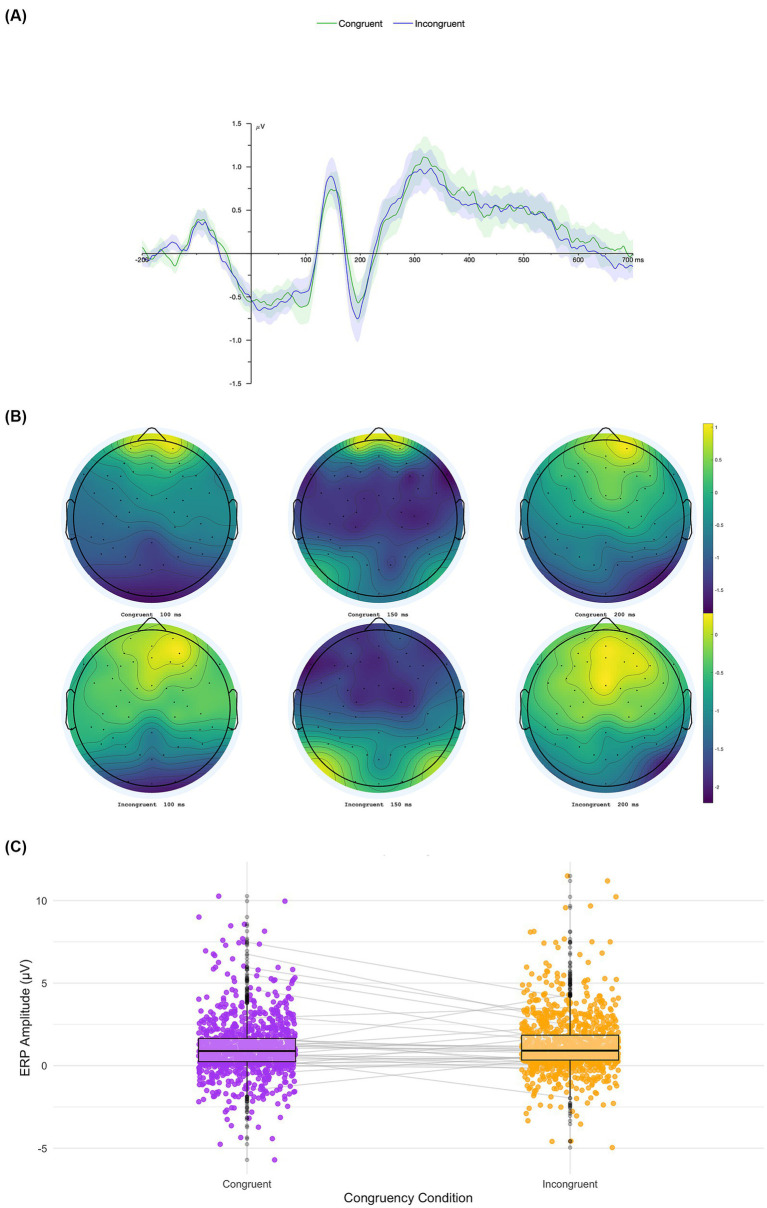
Average waveform and topography of P1 ERP component. Topographical distributions of the P1 component for congruent and incongruent conditions **(A)**, the grand-average ERP waveforms at the selected electrodes **(B)**, and comparatives box plot showing individual subject variability across conditions **(C)**.

We conducted a 3 × 2 ANOVA with Emotional Valence (positive, neutral, negative) and Congruency (congruent vs. incongruent) as within-subjects factors on P1 component amplitudes. The analysis revealed a significant main effect of Congruency, *F*(1, 2,172) = 4.34, *p* = 0.037, *η*^2^ₚ = 0.002 (see Figure 3), indicating that incongruent trials elicited larger P1 amplitudes (*M* = 1.23 μV, *SE* = 0.064) than congruent trials (*M* = 1.09 μV, *SE* = 0.064). No significant main effect of Valence was observed, *F*(2, 2,172) = 0.39, *p* = 0.675, *η*^2^ₚ = 0.0003, nor a significant Congruency × Valence interaction, *F*(2, 2,172) = 0.35, *p* = 0.703, *η*^2^ₚ = 0.0003. *Post-hoc* comparisons with Bonferroni correction confirmed that incongruent trials showed significantly higher amplitudes than congruent trials, *t*(2172) = −2.08, *p* = 0.037, Cohen’s *d* = −0.089, 95% CI [−0.173, −0.005] (see Figure 3).

Amplitudes of the P1 component were consistently higher for incongruent trials across all levels of Valence: positive (congruent: *M* = 1.10, *SE* = 0.26; incongruent: *M* = 1.29, *SE* = 0.27), neutral (congruent: *M* = 1.08, *SE* = 0.24; incongruent: *M* = 1.15, *SE* = 0.24), and negative (congruent: *M* = 1.05, *SE* = 0.21; incongruent: *M* = 1.25, *SE* = 0.21). Descriptive statistics for each condition are presented in Supplementary material.

A verification of statistical assumptions was performed. Homogeneity of variance was confirmed with Levene’s test, *F*(5, 2,172) = 0.67, *p* = 0.643. In addition, normality of the residuals was assessed using the Shapiro–Wilk test, which indicated deviation from normality, *W* = 0.93, *p* < 0.001.

Overall, these results suggest that early P1 component is sensitive to contextual congruency between emotional stimuli, with incongruent cues eliciting enhanced neural responses, regardless of emotional valence. This pattern indicates that the visual system may allocate increased early attentional resources to stimuli that conflict with contextual expectations.

#### 3.3.2 N2 component

**Figure 4:**
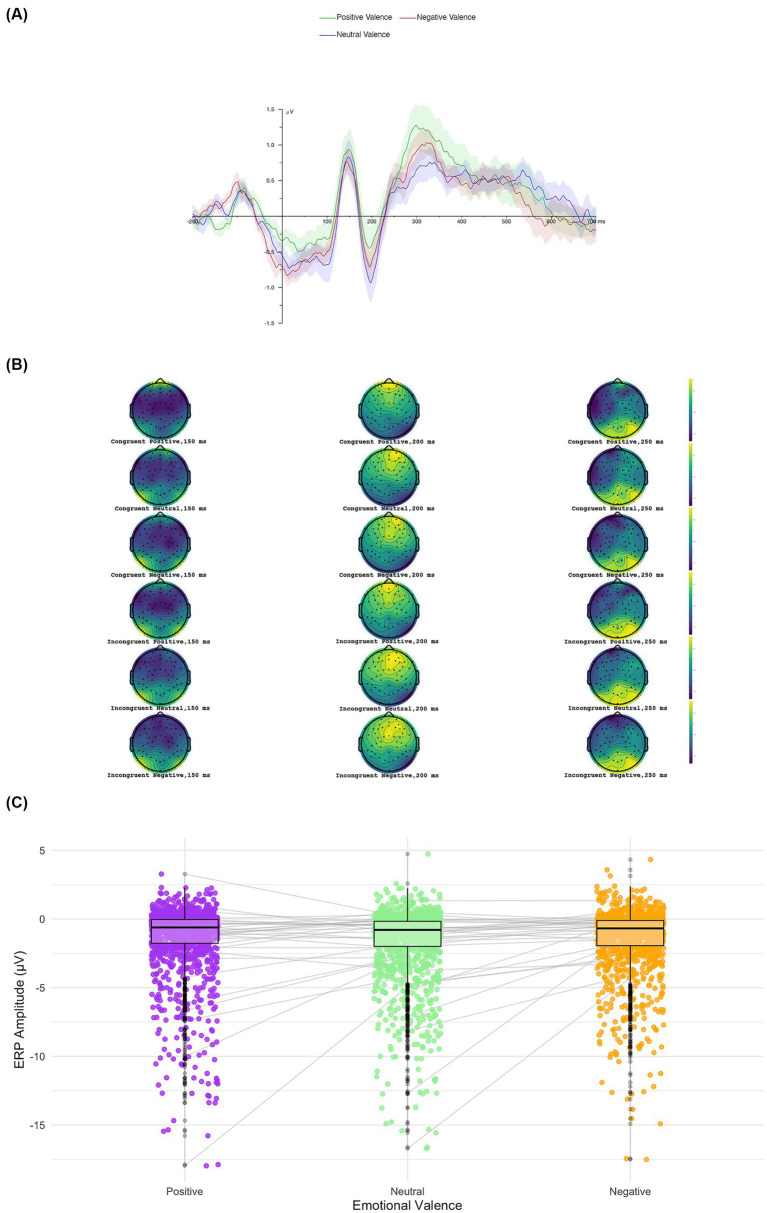
Average waveform and topography of N2 ERP component. Topographical distributions of the N2 component for each valence condition **(A)**, the grand-average ERP waveforms at the selected electrodes **(B)**, and comparative box plot showing individual subject variability across conditions **(C)**.

We conducted a 3 × 2 ANOVA with Emotional Valence (positive, neutral, negative) and Congruency (congruent vs. incongruent) as within-subjects factors on N2 component amplitudes. The analysis revealed a significant main effect of Valence, *F*(2, 3,954) = 3.25, *p* = 0.039, *η*^2^ₚ = 0.002. No significant main effect of Congruency was observed, *F*(1, 3,954) = 2.12, *p* = 0.146, *η*^2^ₚ = 0.0005, nor a significant Congruency × Valence interaction, *F*(2, 3,954) = 0.036, *p* = 0.964, *η*^2^ₚ = 0.00001. However, *post-hoc* comparisons with Bonferroni correction did not confirm differences between emotional valence conditions (see Figure 4). Additionally, pairwise comparisons did not yield significant differences between congruent and incongruent trials considering positive (*p* = 0.331), neutral (*p* = 0.127), or negative (*p* = 0.221) conditions. Similarly, analyses between congruent and incongruent stimuli across valence categories were non-significant after correction (all *p*s > 0.09). Descriptive analyses indicated that N2 amplitudes were consistently negative across conditions (see Supplementary material), with slightly more pronounced negativity for neutral incongruent trials (*M* = −1.71 μV, *SE* = 0.41) compared to congruent neutral trials (*M* = −1.57 μV, *SE* = 0.36).

A verification of statistical assumptions was performed. Homogeneity of variance was confirmed with Levene’s test, *F*(5, 3,954) = 0.87, *p* = 0.499. In addition, normality of the residuals was assessed using the Shapiro–Wilk test, which indicated deviation from normality, *W* = 0.76, *p* < 0.001.

Given the non-significant findings in the standard ANOVA and the importance of ruling out Type II errors (false negatives) in the detection of interaction effects, we conducted a complementary Bayesian ANOVA with default priors in JASP (version 0.19.3; https://jasp-stats.org). We compared the predictive performance of models including the interaction against the Null model. The analysis revealed decisive evidence against the inclusion of the interaction term. Specifically, the Bayes Factor for the full model (Valence + Congruency + Interaction) compared to the Null model was *BF*_10_ = 4.55 × 10^–5^. Conversely, this translates to a *BF*_01_ of approximately 21,978 in favor of the Null hypothesis, indicating that the data are orders of magnitude more likely to occur under a model with no effects than under a model assuming a Valence × Congruency interaction. This suggests that the absence of an N2 interaction effect in our data represents a genuine absence of the effect rather than a lack of statistical sensitivity.

Taken together, the N2 results suggest a trend toward sensitivity to both congruency and emotional valence, but these effects after correction did not reach statistical significance. As shown in Figure 4, which displays the grand-average topographies, ERP waveforms, and subject-level comparative plots, the N2 component exhibited a broadly distributed negativity across conditions, with subtle modulations that were not robust at the congruent or emotional valence level.

#### 3.3.3 P3 component

**Figure 5:**
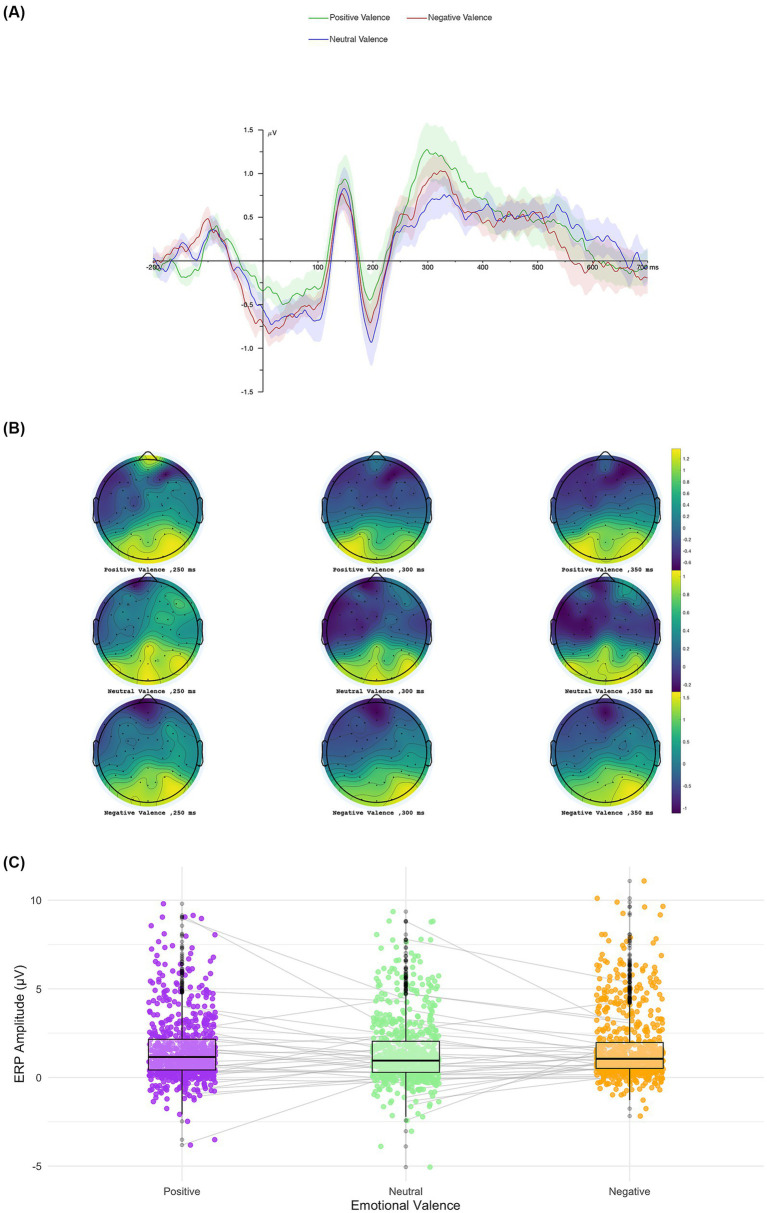
Average waveform and topography of P3 ERP component. Topographical distributions of the P3 component for each valence condition **(A)**, the grand-average ERP waveforms at the selected electrodes **(B)**, and comparatives box plot showing individual subject variability across conditions **(C)**.

We conducted a 3 × 2 ANOVA with Emotional Valence (positive, neutral, negative) and Congruency (congruent vs. incongruent) as within-subjects factors on P3 component amplitudes. The analysis revealed a significant main effect of Valence, *F*(2, 2,172) = 3.08, *p* = 0.046, *η*^2^ₚ = 0.003. No significant main effect of Congruency was observed, *F*(1, 2,172) = 1.60, *p* = 0.205, *η*^2^ₚ = 0.0007, nor a significant Congruency × Valence interaction, *F*(2, 2,172) = 0.043, *p* = 0.958, *η*^2^ₚ = 0.00004. *Post-hoc* comparisons with Bonferroni correction confirmed that P3 component amplitude for negative stimuli (*M* = 1.68 μV, *SE* = 0.070) was significantly larger than for neutral stimuli (*M* = 1.44 μV, *SE* = 0.070), *t*(2172) = 2.48, *p* = 0.040, Cohen’s *d* = 0.13 (see Figure 5). No other *post-hoc* comparisons reached significance (negative vs. positive: *p* = 0.774; positive vs. neutral: *p* = 0.534).

Amplitudes of the P3 component were generally higher for negative stimuli across both congruency conditions: negative (congruent: *M* = 1.73, *SE* = 0.31; incongruent: *M* = 1.64, *SE* = 0.30), neutral (congruent: *M* = 1.48, *SE* = 0.29; incongruent: *M* = 1.40, *SE* = 0.28), and positive (congruent: *M* = 1.64, *SE* = 0.28; incongruent: *M* = 1.50, *SE* = 0.29). Descriptive statistics for each condition are presented in Supplementary material.

A verification of statistical assumptions was performed. Homogeneity of variance was confirmed with Levene’s test, *F*(5, 2,172) = 0.09, *p* = 0.993. In addition, normality of the residuals was assessed using the Shapiro–Wilk test, which indicated deviation from normality, *W* = 0.852, *p* < 0.001.

Overall, these results indicate that the P3 component is modulated by the emotional valence of stimuli, with negative cues eliciting higher neural responses, independent of contextual congruency. This pattern suggests that later stages of cognitive processing may be particularly sensitive to emotionally salient information, reflecting enhanced attentional allocation to negative stimuli.

## 4 Discussion

The present study investigated how contextual congruency between facial and vocal emotional cues shapes emotion recognition at both behavioral and neural levels. By combining reaction time and accuracy measures with ERP recordings, we provide converging evidence that congruency plays a critical role in modulating the temporal dynamics of emotional processing. Our findings indicate that congruent audiovisual combinations facilitated behavioral performance, while incongruent combinations led to longer reaction times and lower accuracy. At the neural level, ERP data revealed that incongruency modulates early processing, as reflected by the P1 component, and that emotional valence modulates later evaluative stages, as indicated by the P3 component. Together, these findings support a multi-stage model for emotional integration, where contextual incongruency impacts processing from early perceptual encoding to sustained cognitive evaluation.

At the behavioral level, the advantage of congruent over incongruent trials is consistent with prior research demonstrating that the matching of audiovisual cues enhances the efficiency of emotion recognition (Föcker et al., 2011; Kreifelts et al., 2007). This congruency effect reflects the ecological reality of everyday communication, where the perception of emotions typically arises from the integration of multimodal cues. The observed impairment in incongruent trials likely reflects a higher cognitive demand to resolve conflicting information, which is consistent with mismatch detection theories (Watson et al., 2013). Specifically, this congruency effect may reflect conflict-driven adjustments in cognitive control (Egner, 2007), where incongruent trials elicit additional recruitment of cognitive control resources. Moreover, social/contextual cues may act as regulators of attentional allocation, guiding processing depending on their relevance and compatibility with the target emotion (Jiménez-Moya et al., 2018). Thus, awareness of (in)congruency may influence both behavioral accuracy and ERP responses, reflecting dynamic adjustments in attentional control. Our findings reinforce the view that congruency is a fundamental organizing principle in the multisensory perception of emotions, which supports adaptive social interactions.

At the neural level, our ERP results reveal the temporal correlates of this congruency effect. We found that incongruent conditions were associated with increased amplitudes in the P1 component, an early wave associated with attentional and sensory processing (Cortés-Rivera and Cerić, 2020). This result, which was contrary to our original hypotheses, suggests that the visual system may allocate heightened early attentional resources to stimuli that conflict with contextual expectations. The P1 modulation by congruency may indicate that conflict detection can occur as early as 100–150 ms after stimulus onset, highlighting the rapid nature of this process. Another explanation is that the enhanced P1 for incongruent cues may reflect increased attentional capture triggered by unexpected or low-probability sensory events (e.g., Dubal et al., 2011). From a multisensory perspective, incongruent audiovisual pairings may therefore lead to heightened sensory gain as the system allocates additional resources to process these unexpected inputs. This interpretation aligns with evidence showing that the P1 is modulated by stimulus salience and expectancy, with larger amplitudes observed when stimuli deviate from contextual predictions or require rapid reorienting of attention.

In interpreting the early ERP results, it is important to note that the P1 component exhibited the expected posterior distribution typically associated with early visual sensory processing. Across conditions, the topographic maps showed maximal activity over occipital and parieto-occipital sites, consistent with prior work indicating that the P1 originates primarily from the extrastriate visual cortex (Clark and Hillyard, 1996; Gomez et al., 1994). No evidence of frontal positivity was observed during the 100–150 ms window, aligning with the well-established view that frontal contributions do not typically emerge at this early latency (Hillyard and Anllo-Vento, 1998; Luck, 2014). Importantly, incongruent trials did not elicit additional frontal engagement that could confound the interpretation of the P1 modulation. Instead, both congruent and incongruent conditions demonstrated a comparable posterior focus, consistent with accounts describing the P1 as a marker of early perceptual encoding rather than frontal-driven processing. This topographic pattern supports the interpretation that the P1 effects observed here reflect enhanced visual–perceptual resource allocation under incongruent contexts, without contamination from frontal sources (Di Russo et al., 2002; Hillyard et al., 1998).

Contrary to our original hypothesis, the congruency modulation of the N2 component did not reach statistical significance. We considered whether this null finding might result from methodological issues, such as a low signal-to-noise ratio (SNR). However, this appears unlikely given the robust significant effects observed for both the preceding P1 and the subsequent P3 components, as well as the high number of trials included to maximize data quality. Instead, the absence of N2 modulation may reflect the specific temporal dynamics of this paradigm. Since robust conflict detection was evident at the earlier P1 stage, it is plausible that the sensory mismatch was resolved during early perceptual encoding, thereby reducing the need for the additional top-down conflict monitoring resources typically indexed by the N2.

Regarding this null finding, we acknowledge that our final sample size for the ERP analysis (*N* = 33), while consistent with standards in the field, warrants consideration regarding statistical power, particularly for detecting subtle within-subject interactions (see Larson and Carbine, 2017). Interaction effects typically require larger samples to detect than main effects, raising the potential concern that our null finding for the N2 component reflects a Type II error (false negative) due to insufficient sensitivity. However, two lines of evidence support the validity of this null result. First, our experimental design prioritized a high number of trials per condition, a factor which has been shown to significantly reduce measurement error and boost the reliability of within-subject comparisons, compensating for moderate sample sizes (Boudewyn et al., 2017). Second, and most importantly, our Bayesian analysis yielded decisive evidence in favor of the null hypothesis (*BF*_01_ > 20,000) regarding the N2 interaction. Unlike frequentist *p*-values, which cannot distinguish between ‘evidence of absence’ and ‘absence of evidence,’ this extreme Bayes Factor indicates that our study was not merely insensitive to the interaction; rather, the data actively contradict the presence of such an effect (Keysers et al., 2020). Therefore, we conclude that the lack of N2 modulation by congruency represents a true negative finding within the context of this paradigm, reinforcing the interpretation that conflict detection for these stimuli is primarily resolved at the earlier P1 stage rather than the later N2 stage.

Our findings also revealed that the P3 component was modulated by the emotional valence of the stimuli, with significantly larger amplitudes for negative stimuli compared to neutral ones. This pattern of results is consistent with the literature highlighting the P3’s role in higher-order cognitive functions, such as stimulus evaluation and the allocation of attention to motivationally significant stimuli (e.g., Balconi and Rovelli, 2024; Liang et al., 2021). The P3 enhancement for negative stimuli, regardless of congruency, suggests that emotional valence is a predominant factor in later stages of evaluative processing. This result aligns with the notion that threatening or negative stimuli preferentially capture and hold attentional resources, which has implications for decision-making and adaptive behavior (see Carretié et al., 2024; Liang et al., 2021).

In the context of Bayesian models and predictive processing frameworks, our findings can be interpreted as reflecting both Bayes-like cue integration, where congruent visual and auditory signals provide more precise combined evidence, and predictive-coding dynamics, where congruent bimodal cues align more closely with the system’s generative predictions, reducing prediction error. Bayesian models of multisensory perception propose that observers combine cues from different modalities by weighting them according to their reliability, often behaving in a Bayes-optimal manner (e.g., Ernst and Banks, 2002). From this perspective, congruent audiovisual trials may facilitate performance because they reduce uncertainty and increase the precision of the sensory evidence available for integration. However, as highlighted by theoretical work (e.g., Harkness and Keshava, 2017), Bayesian models are primarily performance-oriented: they describe how an ideal observer should integrate cues but do not specify the underlying processes or mechanisms. Predictive processing frameworks, in contrast, offer a process-oriented account of how the brain might implement Bayesian inference. Under predictive coding, perception arises from minimizing prediction error through hierarchical generative models in which top-down predictions are continuously compared against bottom-up inputs (Clark, 2013; Hohwy, 2013). In this view, congruent audiovisual stimuli may reduce prediction error at multiple levels of the hierarchy, thereby facilitating faster or more accurate responses.

Despite the contributions of this study, some limitations must be considered. First, the experimental design utilized congruent bimodal stimuli (visual and auditory) presented simultaneously. While this approach enhances ecological validity, it limits the ability to disentangle the specific contribution of each modality to the observed effects. Since the study did not include unimodal control conditions (i.e., auditory-only or visual-only trials), it is not possible to quantify the absolute magnitude of multisensory gain or to isolate the independent contribution of each sensory modality to the behavioral response. However, the significant main effects of congruency and the interaction between valence and congruency suggest that the results derive from the integration of both modalities rather than the isolated processing of a single component. Second, the results are limited to a neurotypical population, which restricts the generalizability of the findings to clinical populations. Future research could replicate this study including unimodal baselines to further clarify the specific role of auditory and visual inputs in bimodal processing, and extend the paradigm to populations with known deficits in emotion recognition, such as individuals with autism or mood disorders. Furthermore, the use of more complex contextual cues, such as social narratives or dynamic environments, could provide a more nuanced understanding of multisensory emotion integration in real-life scenarios. Finally, the integration of other neuroimaging modalities, such as fMRI, could help to elucidate the underlying neural networks for the observed congruency and valence effects.

Finally, although age and gender were not included as factors in the main analyses—given that these variables were not central to the aims of the present study—we conducted exploratory checks to ensure they did not confound the behavioral results. Consistent with our hypotheses, we did not observe significant effects of age or gender on reaction times or accuracy. Nonetheless, emerging evidence suggests that sociodemographic factors may influence neural responses in certain contexts (e.g., Pua and Yu, 2024; Yener et al., 2024). Therefore, we acknowledge the absence of these variables in our ERP models as a limitation and recommend that future research examine the extent to which age, gender, and other demographic characteristics may moderate early neural markers of multisensory processing.

In addition to the ERP components analyzed in this study, it is worth noting that other neural markers such as the N400 and the late positive potential (LPP) have been associated with semantic incongruency and sustained emotional evaluation (Aguado et al., 2013; Brown et al., 2012; Kutas and Federmeier, 2011; Liu et al., 2012). However, the present paradigm was intentionally designed to capture rapid multisensory congruency detection and short-latency affective responses. The brief stimulus presentations and fast trial dynamics emphasize early perceptual encoding and mid-latency evaluative processes rather than the extended semantic integration or prolonged affective processing typically required to elicit robust N400 or LPP activity. For these methodological reasons, the analysis focused *a priori* on the P1, N2, and P3 components, which are theoretically aligned with the temporal stages targeted by the task. Future research employing longer stimulus exposures or explicit semantic manipulations may help clarify whether later components such as the N400 or LPP also contribute to contextual emotional integration in similar paradigms.

In summary, our findings support a multi-stage model for the integration of emotional cues, where early processing is driven by contextual congruency, and later processing is modulated by emotional valence. This study clarifies the temporal course of contextual integration in the multisensory perception of emotions, providing new insights with implications for research in both affective and cognitive neuroscience.

## Data Availability

The datasets presented in this study can be found in online repositories. The names of the repository/repositories and accession number(s) can be found at: https://osf.io/fuhj7/?view_only=3d3ced4c448e42088a368a12ebe7ea79.
