# Supplementary material for: Contextual cues shape facial emotion recognition: a combined behavioral and ERP study
Source: Front Neurosci. 2026 Jan 14;19:1710208. doi: 10.3389/fnins.2025.1710208 (PMC12847258; doi:10.3389/fnins.2025.1710208)
Supplement: Supplementary file 1 [file Table_1.docx]

***Supplementary Material***

Supplementary Material of the article entitled: **“Contextual Cues Shape Facial Emotion Recognition: A Combined Behavioral and ERP Study”**.

# Supplementary Tables

# 1.1 Descriptive Statistics

**1.1.1 Participants characteristics and self-reported scores grouped by sex**

| **Instrument** | **Sex** | **N** | **Mean** | **Median** | **SD** | **Minimum** | **Maximum** |
| --- | --- | --- | --- | --- | --- | --- | --- |
| PHQ-9 | Men | 20 | 4.15 | 3.5 | 3.54 | 0 | 12 |
|  | Women | 19 | 4.79 | 4.0 | 4.05 | 0 | 13 |
| PANAS | Men | 20 | 18.7 | 22.5 | 10.15 | 0 | 32 |
|  | Women | 19 | 15.16 | 12.0 | 13.5 | -17 | 38 |
| Stai-S | Men | 20 | 25.9 | 26.5 | 4.7 | 19 | 34 |
|  | Women | 19 | 29.16 | 26.0 | 9.82 | 16 | 60 |
| Beck | Men | 20 | 7.05 | 4.5 | 6.27 | 0 | 19 |
|  | Women | 19 | 6.37 | 5.0 | 4.36 | 2 | 17 |

**1.1.1 Participants characteristics and self-reported scores grouped by age**

| **Instrument** | **Age** | **N** | **Mean** | **Median** | **SD** | **Minimum** | **Maximum** |
| --- | --- | --- | --- | --- | --- | --- | --- |
| PANAS | 18 | 1 | 5.00 | 5 | NaN | 5 | 5 |
|  | 19 | 7 | 16.71 | 19 | 9.286 | 5 | 29 |
|  | 20 | 2 | 20.00 | 20.00 | 12.728 | 11 | 29 |
|  | 21 | 2 | 13.50 | 13.50 | 0.707 | 13 | 14 |
|  | 22 | 4 | 4.50 | 5.00 | 17.635 | -17 | 25 |
|  | 24 | 1 | 5.00 | 5 | NaN | 5 | 5 |
|  | 25 | 3 | 17.67 | 23 | 13.796 | 2 | 28 |
|  | 27 | 1 | 19.00 | 19 | NaN | 19 | 19 |
|  | 29 | 1 | 32.00 | 32 | NaN | 32 | 32 |
|  | 30 | 2 | 11.50 | 11.50 | 0.707 | 11 | 12 |
|  | 31 | 3 | 22.67 | 23 | 0.577 | 22 | 23 |
|  | 32 | 2 | 28.00 | 28.00 | 0.000 | 28 | 28 |
|  | 33 | 1 | 25.00 | 25 | NaN | 25 | 25 |
|  | 35 | 4 | 12.25 | 9.50 | 11.955 | 2 | 28 |
|  | 37 | 1 | 31.00 | 31 | NaN | 31 | 31 |
|  | 38 | 2 | 19.00 | 19.00 | 26.870 | 0 | 38 |
|  | 39 | 1 | 32.00 | 32 | NaN | 32 | 32 |
|  | 40 | 1 | 24.00 | 24 | NaN | 24 | 24 |
| Stai-S | 18 | 1 | 25.00 | 25 | NaN | 25 | 25 |
|  | 19 | 7 | 28.86 | 32 | 6.669 | 20 | 37 |
|  | 20 | 2 | 26.00 | 26.00 | 1.414 | 25 | 27 |
|  | 21 | 2 | 30.50 | 30.50 | 4.950 | 27 | 34 |
|  | 22 | 4 | 34.50 | 27.00 | 17.078 | 24 | 60 |
|  | 24 | 1 | 20.00 | 20 | NaN | 20 | 20 |
|  | 25 | 3 | 23.67 | 22 | 2.887 | 22 | 27 |
|  | 27 | 1 | 23.00 | 23 | NaN | 23 | 23 |
|  | 29 | 1 | 21.00 | 21 | NaN | 21 | 21 |
|  | 30 | 2 | 29.00 | 29.00 | 2.828 | 27 | 31 |
|  | 31 | 3 | 27.33 | 31 | 7.234 | 19 | 32 |
|  | 32 | 2 | 25.50 | 25.50 | 6.364 | 21 | 30 |
|  | 33 | 1 | 31.00 | 31 | NaN | 31 | 31 |
|  | 35 | 4 | 28.50 | 27.00 | 9.292 | 19 | 41 |
|  | 37 | 1 | 23.00 | 23 | NaN | 23 | 23 |
|  | 38 | 2 | 25.50 | 25.50 | 13.435 | 16 | 35 |
|  | 39 | 1 | 26.00 | 26 | NaN | 26 | 26 |
|  | 40 | 1 | 23.00 | 23 | NaN | 23 | 23 |
| Beck | 18 | 1 | 7.00 | 7 | NaN | 7 | 7 |
|  | 19 | 7 | 5.14 | 5 | 3.532 | 0 | 11 |
|  | 20 | 2 | 3.00 | 3.00 | 1.414 | 2 | 4 |
|  | 21 | 2 | 10.50 | 10.50 | 2.121 | 9 | 12 |
|  | 22 | 4 | 10.50 | 10.00 | 8.699 | 3 | 19 |
|  | 24 | 1 | 5.00 | 5 | NaN | 5 | 5 |
|  | 25 | 3 | 6.00 | 3 | 7.000 | 1 | 14 |
|  | 27 | 1 | 5.00 | 5 | NaN | 5 | 5 |
|  | 29 | 1 | 2.00 | 2 | NaN | 2 | 2 |
|  | 30 | 2 | 9.50 | 9.50 | 3.536 | 7 | 12 |
|  | 31 | 3 | 4.67 | 5 | 3.512 | 1 | 8 |
|  | 32 | 2 | 1.00 | 1.00 | 1.414 | 0 | 2 |
|  | 33 | 1 | 3.00 | 3 | NaN | 3 | 3 |
|  | 35 | 4 | 8.75 | 7.50 | 5.123 | 4 | 16 |
|  | 37 | 1 | 3.00 | 3 | NaN | 3 | 3 |
|  | 38 | 2 | 10.50 | 10.50 | 9.192 | 4 | 17 |
|  | 39 | 1 | 4.00 | 4 | NaN | 4 | 4 |
|  | 40 | 1 | 19.00 | 19 | NaN | 19 | 19 |
| PHQ-9 | 18 | 1 | 12.00 | 12 | NaN | 12 | 12 |
|  | 19 | 7 | 5.71 | 3 | 5.090 | 0 | 13 |
|  | 20 | 2 | 3.00 | 3.00 | 4.243 | 0 | 6 |
|  | 21 | 2 | 7.00 | 7.00 | 5.657 | 3 | 11 |
|  | 22 | 4 | 5.00 | 4.00 | 5.099 | 0 | 12 |
|  | 24 | 1 | 4.00 | 4 | NaN | 4 | 4 |
|  | 25 | 3 | 4.33 | 3 | 4.163 | 1 | 9 |
|  | 27 | 1 | 4.00 | 4 | NaN | 4 | 4 |
|  | 29 | 1 | 4.00 | 4 | NaN | 4 | 4 |
|  | 30 | 2 | 6.50 | 6.50 | 2.121 | 5 | 8 |
|  | 31 | 3 | 3.00 | 3 | 1.000 | 2 | 4 |
|  | 32 | 2 | 1.00 | 1.00 | 1.414 | 0 | 2 |
|  | 33 | 1 | 3.00 | 3 | NaN | 3 | 3 |
|  | 35 | 4 | 4.75 | 5.50 | 2.630 | 1 | 7 |
|  | 37 | 1 | 0.00 | 0 | NaN | 0 | 0 |
|  | 38 | 2 | 3.00 | 3.00 | 4.243 | 0 | 6 |
|  | 39 | 1 | 0.00 | 0 | NaN | 0 | 0 |
|  | 40 | 1 | 5.00 | 5 | NaN | 5 | 5 |
