# Supplementary material for: Contextual cues shape facial emotion recognition: a combined behavioral and ERP study
Source: Front Neurosci. 2026 Jan 14;19:1710208. doi: 10.3389/fnins.2025.1710208 (PMC12847258; doi:10.3389/fnins.2025.1710208)
Supplement: Supplementary file 2 [file Table_2.docx]

***Supplementary Material***

Supplementary Material of the article entitled: **“Contextual Cues Shape Facial Emotion Recognition: A Combined Behavioral and ERP Study”**.

# Supplementary Tables

# 1.1 Descriptive Statistics

**1.1.2 Verification of assumptions and** **Mann–Whitney U test for screening variables by participant sex**

| **Screening Variable** | **W** | **p** | **F** | **df1** | **df2** | **p** |
| --- | --- | --- | --- | --- | --- | --- |
| Age | .904 | .003 | 0.0204 | 1 | 37 | .887 |
| BDI-II | .892 | .001 | 4.474 | 1 | 37 | .041 |
| STAI-S | .871 | <.001 | 4.236 | 1 | 37 | .047 |
| PANAS | .971 | .392 | 1.382 | 1 | 37 | .247 |
| PHQ-9 | .910 | .004 | 0.381 | 1 | 37 | .541 |

| **Screening Variable** | **U** | ***p*** |
| --- | --- | --- |
| Age | 164 | .472 |
| BDI-II | 180 | .789 |
| STAI-S | 156 | .345 |
| PANAS | 162 | .439 |
| PHQ-9 | 175 | .671 |

*Note*. U = Mann–Whitney U test fo independent samples.
