# Supplementary material for: Contextual cues shape facial emotion recognition: a combined behavioral and ERP study
Source: Front Neurosci. 2026 Jan 14;19:1710208. doi: 10.3389/fnins.2025.1710208 (PMC12847258; doi:10.3389/fnins.2025.1710208)
Supplement: Supplementary file 3 [file Table_3.docx]

***Supplementary Material***

Supplementary Material of the article entitled: **“Contextual Cues Shape Facial Emotion Recognition: A Combined Behavioral and ERP Study”**.

# Supplementary Tables

# 1.1 Descriptive Statistics

**1.1.3 Amplitude of the P1 component organized by condition**

|  | **Congruency Condition** | **Valence Condition** | **N** | **Mean** | **Median** | **SD** | **Minimum** | **Maximum** |
| --- | --- | --- | --- | --- | --- | --- | --- | --- |
| Amplitude  (μV) | Congruent | Negative | 363 | 1.05 | 0.826 | 1.53 | -2.80 | 9.00 |
|  |  | Neutral | 363 | 1.08 | 0.886 | 1.69 | -4.76 | 9.96 |
|  |  | Positive | 363 | 1.1 | 0.943 | 1.76 | -5.71 | 10.26 |
|  | Incongruent | Negative | 363 | 1.25 | 0.857 | 1.55 | -3.34 | 10.22 |
|  |  | Neutral | 363 | 1.15 | 0.799 | 1.69 | -3.54 | 11.19 |
|  |  | Positive | 363 | 1.29 | 0.974 | 1.79 | -4.96 | 11.49 |
