# Supplementary material for: Contextual cues shape facial emotion recognition: a combined behavioral and ERP study
Source: Front Neurosci. 2026 Jan 14;19:1710208. doi: 10.3389/fnins.2025.1710208 (PMC12847258; doi:10.3389/fnins.2025.1710208)
Supplement: Supplementary file 4 [file Table_4.docx]

***Supplementary Material***

Supplementary Material of the article entitled: **“Contextual Cues Shape Facial Emotion Recognition: A Combined Behavioral and ERP Study”**.

# Supplementary Tables

# 1.1 Descriptive Statistics

**1.1.4 Amplitude of the N2 component organized by condition**

|  | **Congruency Condition** | **Valence Condition** | **N** | **Mean** | **Median** | **SD** | **Minimum** | **Maximum** |
| --- | --- | --- | --- | --- | --- | --- | --- | --- |
| Amplitude  (μV) | Congruent | Negative | 660 | -1.38 | -0.690 | 2.27 | -17.4 | 2.38 |
|  |  | Neutral | 660 | -1.57 | -0.754 | 2.47 | -16.6 | 4.73 |
|  |  | Positive | 660 | -1.36 | -0.635 | 2.45 | -17.9 | 2.12 |
|  | Incongruent | Negative | 660 | -1.49 | -0.686 | 2.52 | -17.5 | 4.32 |
|  |  | Neutral | 660 | -1.71 | -0.848 | 2.75 | -16.7 | 2.21 |
|  |  | Positive | 660 | -1.46 | -0.603 | 2.74 | -18.0 | 3.27 |
