# Supplementary material for: Contextual cues shape facial emotion recognition: a combined behavioral and ERP study
Source: Front Neurosci. 2026 Jan 14;19:1710208. doi: 10.3389/fnins.2025.1710208 (PMC12847258; doi:10.3389/fnins.2025.1710208)
Supplement: Supplementary file 5 [file Table_5.docx]

***Supplementary Material***

Supplementary Material of the article entitled: **“Contextual Cues Shape Facial Emotion Recognition: A Combined Behavioral and ERP Study”**.

# Supplementary Tables

# 1.1 Descriptive Statistics

**1.1.5 Amplitude of the P3 component organized by condition**

|  | **Congruency Condition** | **Valence Condition** | **N** | **Mean** | **Median** | **SD** | **Minimum** | **Maximum** |
| --- | --- | --- | --- | --- | --- | --- | --- | --- |
| Amplitude  (μV) | Congruent | Negative | 363 | 1.73 | 1.073 | 1.96 | -1.76 | 11.09 |
|  |  | Neutral | 363 | 1.48 | 0.978 | 1.88 | -5.06 | 9.36 |
|  |  | Positive | 363 | 1.64 | 1.232 | 1.82 | -2.48 | 9.80 |
|  | Incongruent | Negative | 363 | 1.64 | 1.004 | 1.89 | -2.18 | 10.11 |
|  |  | Neutral | 363 | 1.40 | 0.941 | 1.83 | -3.89 | 8.82 |
|  |  | Positive | 363 | 1.50 | 0.989 | 1.87 | -3.81 | 9.14 |
